# Rippled β-Sheet Formation by an Amyloid-β Fragment Indicates Expanded Scope of Sequence Space for Enantiomeric β-Sheet Peptide Coassembly

**DOI:** 10.3390/molecules24101983

**Published:** 2019-05-23

**Authors:** Jennifer M. Urban, Janson Ho, Gavin Piester, Riqiang Fu, Bradley L. Nilsson

**Affiliations:** 1 Department of Chemistry, University of Rochester, Rochester, NY 14627-0216, USA; jurban4@ur.rochester.edu (J.M.U.); janson.ho1@gmail.com (J.H.); gpiester@u.rochester.edu (G.P.); 2 The National High Magnetic Field Laboratory, Florida State University, 1800 East Paul Dirac Drive, Tallahassee, FL 32310, USA; rfu@magnet.fsu.edu

**Keywords:** peptide coassembly, rippled β-sheets, amphipathic peptides, enantiomeric coassembly

## Abstract

In 1953, Pauling and Corey predicted that enantiomeric β-sheet peptides would coassemble into so-called “rippled” β-sheets, in which the β-sheets would consist of alternating l- and d-peptides. To date, this phenomenon has been investigated primarily with amphipathic peptide sequences composed of alternating hydrophilic and hydrophobic amino acid residues. Here, we show that enantiomers of a fragment of the amyloid-β (Aβ) peptide that does not follow this sequence pattern, amyloid-β (16–22), readily coassembles into rippled β-sheets. Equimolar mixtures of enantiomeric amyloid-β (16–22) peptides assemble into supramolecular structures that exhibit distinct morphologies from those observed by self-assembly of the single enantiomer pleated β-sheet fibrils. Formation of rippled β-sheets composed of alternating l- and d-amyloid-β (16–22) is confirmed by isotope-edited infrared spectroscopy and solid-state NMR spectroscopy. Sedimentation analysis reveals that rippled β-sheet formation by l- and d-amyloid-β (16–22) is energetically favorable relative to self-assembly into corresponding pleated β-sheets. This work illustrates that coassembly of enantiomeric β-sheet peptides into rippled β-sheets is not limited to peptides with alternating hydrophobic/hydrophilic sequence patterns, but that a broader range of sequence space is available for the design and preparation of rippled β-sheet materials.

## 1. Introduction

Cross-β amyloid fibrils are β-sheet rich supramolecular assemblies formed by a wide variety of peptides and proteins. The unregulated self-assembly of peptides and proteins into amyloid aggregates is characteristic of protein misfolding pathologies that notably include Alzheimer’s disease, Parkinson’s disease, and prion encephalopathies [1,2,3,4]. Amyloid fibrils have also evolved as regulated functional materials in biological systems [5,6,7,8,9]. These materials have inspired extensive efforts in the development of novel supramolecular biomaterials composed of peptides that self-assemble into amyloid or amyloid-like architectures with engineered emergent properties [10,11,12].

**Figure 1:**
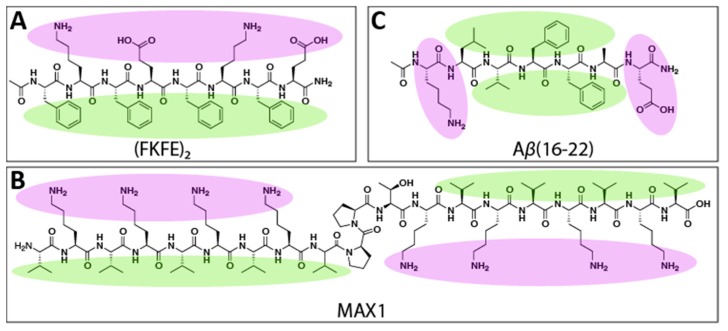
Structures of (**A**). Ac-(FKFE)_2_-NH_2_, (**B**). MAX1, and (**C**). Aβ(16–22) with the nonpolar side chains highlighted in green and polar side chains in purple.

Rippled β-sheets are an emerging class of amyloid-inspired material. In 1953, Pauling and Corey predicted that enantiomeric β-sheet peptides would selectively coassemble into so-called “rippled β-sheets” composed of alternating l- and d-peptides [13]. These two-component assemblies were expected to adopt a rippled appearance, as opposed to the pleated structure of single-enantiomer β-sheets. One of the earliest experimental examples of enantiomeric peptide coassembly was of mixtures of poly(l-lysine) and poly(d-lysine), though the resulting assemblies were only recently proven to be composed of racemic β-sheets [14,15]. Similarly, mixtures of poly(l-glutamic acid) and poly(d-glutamic acid) have been found to coassemble into amyloid-like fibrils, albeit slowly and with many defects [16]. Enantiomeric amphipathic peptides with alternating polar/nonpolar amino acid sequence patterns have been recently shown to coassemble into rippled β-sheet materials [17,18,19,20]. For example, we discovered that mixtures of l- and d-Ac-(FKFE)_2_-NH_2_ peptides (Figure 1A), which readily self-assemble into bilayer nanoribbon fibrils, preferentially coassemble into rippled β-sheets in which the l- and d-peptides alternate with high fidelity. The coassembled rippled β-sheets were found to be energetically more stable than the corresponding one-component pleated β-sheet assemblies [17,18]. Schneider and coworkers also found that enantiomers of the MAX1 peptide (Figure 1B), a β-hairpin peptide that self-assembles into β-sheet bilayer fibrils that form hydrogel networks, also coassemble into rippled β-sheet fibrils [19,20]. Furthermore, hydrogels formed from coassembled l- and d-Ac-(FKFE)_2_-NH_2_ and l- and d-MAX1 were found to be mechanically stronger than those formed from a single enantiomer [17,20]. Recent structural analysis of the MAX1 rippled β-sheets shows that the enhanced viscoelasticity of the rippled β-sheet relative to the pleated β-sheet hydrogel networks is due to the distinctive rippled structure of the two-component fibrils [19].

These rippled β-sheets identified by Schneider and Nilsson are formed by similar peptides with alternating polar and nonpolar amino acid sequences. This raises the question as to whether the coassembly of enantiomeric peptides into rippled β-sheets is unique to this class of self-assembling peptide or if rippled β-sheets can be formed generally by any self-assembling β-strand peptide regardless of sequence pattern. In this study, we investigate whether enantiomeric peptide coassembly into rippled β-sheets can be extended to a peptide that does not display alternating polar/nonpolar sidechains by studying the coassembly propensity of enantiomers of a well-studied self-assembling fragment of the Alzheimer’s disease amyloid-β peptide, Aβ(16–22) (Ac-KLVFFAE-NH_2_, Figure 1C). Comprising the hydrophobic core of amyloid-β, the residues 16–22 are believed to be critical in the assembly mechanism of the full-length peptide. The 16–22 residue fragment readily self-assembles into amyloid-like fibrils reminiscent of those formed by the parent peptide, and as such, Aβ(16–22) has served as a model peptide in many investigations into amyloid peptide self-assembly [21,22,23,24,25,26,27]. The Aβ(16–22) peptide lacks the alternating polar/nonpolar amino acid sequence pattern common to Ac-(FKFE)_2_-NH_2_ and MAX1. Instead, the polar residues of Aβ(16–22) are isolated at the *N*- and *C*-termini, with the intervening amino acids comprising a nonpolar core (as shown in Figure 1C). We anticipated that an interrogation of the coassembly propensity of the l- and d-enantiomers of Aβ(16–22) would provide insight into the possibility that rippled β-sheet assembly may be a general property of all β-sheet peptides, as predicted by Pauling and Corey.

## 2. Results and Discussion

**Figure 2:**
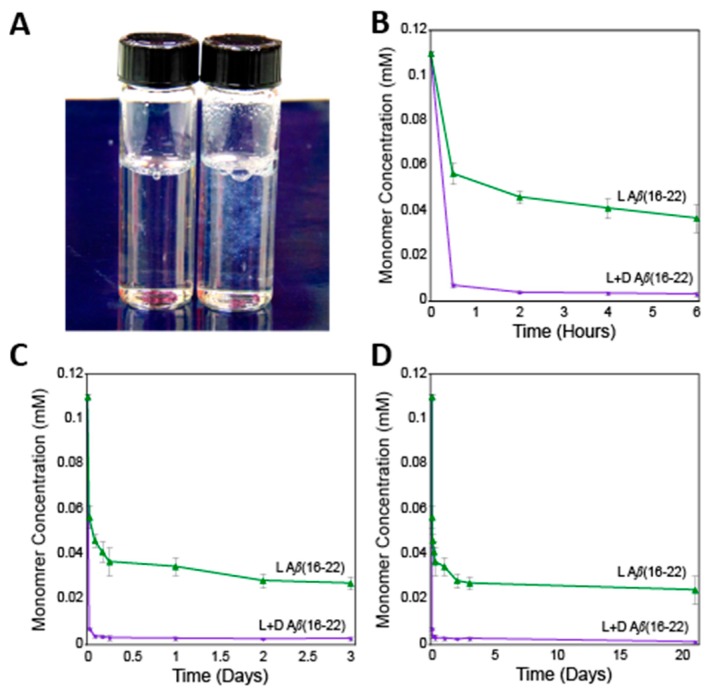
(**A**). Digital image of L-Aβ(16–22) self-assembly (left) and l/d-Aβ(16–22) coassembly (right) immediately after mixing. (**B**). Sedimentation data over six hours. (**C**). Sedimentation data over three days. (**D**). Sedimentation data over 21 days.

The self-assembly of l- and d-Ac-KLVFFAE-NH_2_ peptides was compared to the assembly properties of an equimolar mixture of the enantiomers. The peptides were prepared via standard Fmoc solid phase peptide synthesis, purified by reverse-phase HPLC, and characterized by MALDI-TOF mass spectrometry (see Materials and Methods section for experimental details and Supplementary Materials for characterization and purity data). Prior to self-assembly, peptides were disaggregated by sequential treatment with trifluoroacetic acid and hexafluoroisopropanol using a modified Wetzel protocol [28]; peptide concentrations were determined by comparison to standard analytical HPLC curves (see Supplementary Materials for details), as described previously for studies with Aβ(16–22) [22,23]. Disaggregated, lyophilized peptides were then dissolved in DMSO and diluted into phosphate-buffered saline (pH 7.4) to reach total peptide concentrations of 110 μM (55 μM of each l- and d-enantiomer for mixed coassembly studies) (final DMSO concentration was 5% *v*/*v*). Upon dilution, peptides were incubated at 37 °C for assembly. Immediately after gentle mixing, visible precipitate was observed in the l/d mixed samples, but not in the single enantiomer self-assembly controls (see Figure 2A), indicating much more rapid assembly in the mixed enantiomer samples consistent with rippled β-sheet formation.

### 2.1. Sedimentation Analysis

Comparison of self-assembly and coassembly of Aβ(16–22) was conducted by a time-dependent sedimentation analysis. For these studies, we adapted Wetzel’s HPLC sedimentation protocol [28] that we have previously used to assess Aβ(16–22) self-assembly [22,23]. Aliquots of self-assembly and coassembly mixtures were removed over time and subjected to ultracentrifugation to remove fibrils and lower order aggregates after which the concentration of unaggregated peptide was quantified. In these analyses, equilibrium between monomer and fibril is eventually reached, with the final monomer concentration, or critical concentration (*C_r_*), providing a quantitative characterization of the dynamic equilibrium between fibrils of length *n* and monomer with fibrils of length *n* + 1. This *C_r_* value is inversely related to the association constant for the addition of monomer to fibril, *K_a_*, as shown in Equation (1). The *K_a_* value can be used to determine the relative free energy, ΔG, for addition of monomer to fibril at equilibrium [28].

(1) $$C_{r}=\frac{\left[fibril_{n}\right]\left[\mathrm{monomer}\right]}{\left[fibril_{n+1}\right]}=\frac{1}{K_{a}}$$

Sedimentation analysis revealed distinctive kinetic and thermodynamic properties for the pleated β-sheet self-assembly of single enantiomers of Aβ(16–22) compared to rippled β-sheet coassembly of the mixed enantiomers. The l/d-Aβ(16–22) mixture showed a rapid decrease in monomer concentration within 30 min of mixing (Figure 2B–D). After six hours, the coassembly sample had essentially reached equilibrium (Figure 2B). In contrast, the single enantiomer self-assembly samples required over two weeks to reach a constant monomer concentration (Figure 2C,D). The self-assembled single enantiomer Aβ(16–22) sample had a *C_r_* of 24 ± 6 μM, corresponding to a ΔG value of −6.6 ± 0.2 kcal mol^−1^, consistent with our previously reported data [22,23]. Samples containing 1:1 l:d Aβ(16–22) had a significantly lower *C_r_* of 1.1 ± 0.8 μM with a corresponding ΔG of −8.4 ± 0.4 kcal mol^−1^. Thus, rippled β-sheet coassembly has a ΔΔG of −1.8 kcal mol^−1^ relative to pleated β-sheet self-assembly of the l- or d-enantiomers alone, revealing a significant enhancement in thermodynamic stability for the coassembled rippled β-sheet Aβ(16–22) aggregates. This is consistent with the enhanced thermodynamic stability of rippled β-sheets of the amphipathic Ac-(FKFE)_2_-NH_2_ peptide [18]. Note, complementary thioflavin T (ThT) fluorescence assays were not performed in addition to the sedimentation analysis due to the literature precedent of low binding affinity and low fluorescence response of ThT to Aβ(16–22) aggregates [23,29,30,31,32].

### 2.2. Transmission and Scanning Electron Microscopy

**Figure 3:**
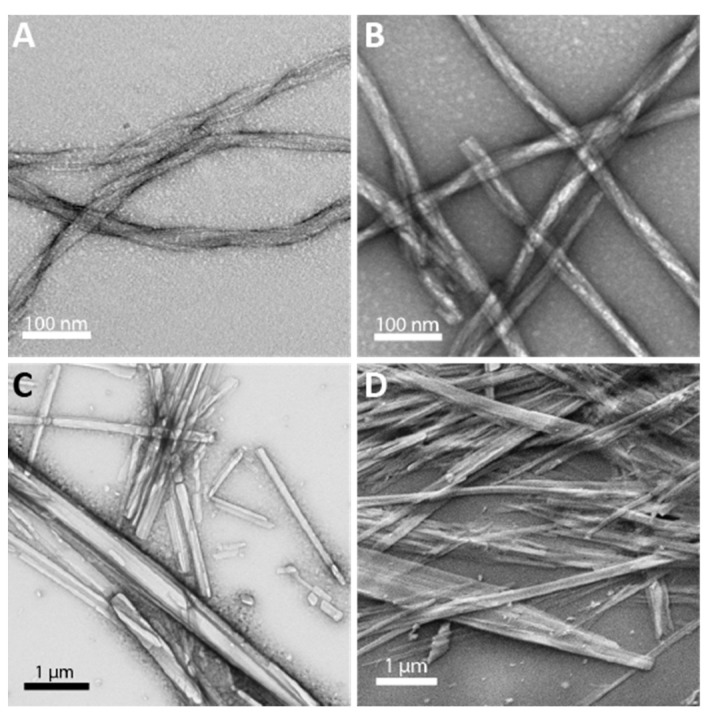
Transmission electron micrographs of assemblies formed by (**A**). l-Aβ(16–22), (**B**). d-Aβ(16–22), and (**C**). l/d-Aβ(16–22). (**D**). Scanning electron micrograph of assemblies formed by l/d-Aβ(16–22).

The morphology of the self-assembled and coassembled materials was compared by transmission electron microscopy (TEM). As shown in Figure 3A,B, the l- and d-single enantiomer, self-assembled, pleated β-sheets formed flexible twisted fibrils with widths of 11.8 ± 0.5 nm, consistent with previous reports [22,23]. However, the mixed l/d aggregates exhibited dramatically different morphologies that were rigid with a semi-crystalline appearance, having widths ranging from 43−593 nm (Figure 3C). Scanning electron microscopy (SEM) confirmed this unique morphology for the coassembly structures (Figure 3D). While these putative rippled β-sheet assemblies appear semi-crystalline in microscopic images, they lacked sufficient dimensionality for single crystal X-ray diffraction (see Figure 3D). The morphologies of each sample were very reproducible and did not change over time. Once fibrils form in the l- and d-single enantiomer samples (several days), no changes were seen over 21 days. Immediately after dissolution, the mixed l/d aggregates appear as rigid, semi-crystalline structures with no major changes over 21 days. The distinctive morphology of the l/d aggregates of Aβ(16–22) is consistent with the unique morphologies observed for nanofibrils of mixed enantiomer Ac-(FKFE)_2_-NH_2_ rippled β-sheets compared to their self-assembled counterparts, providing further evidence that Aβ(16–22) enantiomers also assemble into rippled β-sheet structures [18].

### 2.3. Isotope Edited Infrared Spectroscopy

**Figure 4:**
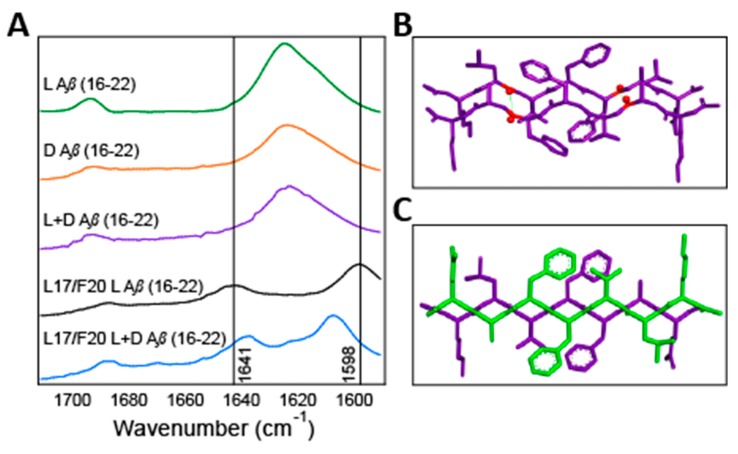
(**A**). Fourier Transform Infrared Spectroscopy (FTIR) overlays of self-assembled l- and d-Aβ(16–22), coassembled l/d-Aβ(16–22), ^13^C labeled l-Aβ(16–22), and coassembled ^13^C l-/ unlabeled d-Aβ(16–22). (**B**). A structural model for putative pleated β-sheets of self-assembled l-Aβ(16–22) with ^13^C labeled positions highlighted in red. (**C**). A proposed structural model l/d-Aβ(16–22) coassembled rippled β-sheets.

In order to confirm that the l- and d-enantiomers of Aβ(16–22) were indeed packing into alternating l/d rippled β-sheets, we conducted isotope-edited infrared (IE-IR) spectroscopy studies. Antiparallel β-sheets have characteristic IR amide I stretching frequencies at 1624 and 1690 cm^−1^, as was seen for the assemblies of l-Aβ(16–22), d-Aβ(16–22), and l/d-Aβ(16–22) (Figure 4A). Isotope edited IR (IE-IR) was used to further our understanding of these assemblies. To this end, we synthesized l-Aβ(16–22) with ^13^C labels at the carbonyls of L17 and F20. When this labeled peptide self-assembles, these heavier atoms are in close proximity and coupled (see Figure 4B), resulting in the shifting and splitting of the characteristic β-sheet peak at 1624 cm^−1^ to 1641 cm^−1^ and 1598 cm^−1^ (Figure 4A, black trace). Next, this labeled l-Aβ(16–22) was mixed with an equimolar amount of unlabeled d-Aβ(16–22). If the enantiomeric strands were self-sorting, we would expect these 1641 cm^−1^ and 1598 cm^−1^ signals to be preserved because the cross-strand coupling would not be interrupted. However, we saw instead a shift of these signals to 1638 cm^−1^ and 1608 cm^−1^ with a corresponding smaller splitting (Figure 4A, blue trace). This 10 cm^−1^ shift in the higher energy peak from 1598 cm^−1^ to 1608 cm^−1^ indicates that the unlabeled d-Aβ(16–22) is in a cross-strand packing arrangement with the labeled l-Aβ(16–22) peptide such that it interrupts the coupling between the labeled carbonyls. Similar shifts of 9 to 10 cm^−1^ in other self-assembled systems clearly indicate changes in coupling state, thus these data indicate that enantiomers of Aβ(16–22) also coassemble into Pauling and Corey’s rippled β-sheets with alternating l- and d-strands as modeled in Figure 4C [18,19].

### 2.4. Solid-State Nuclear Magnetic Resonance Spectroscopy

**Figure 5:**
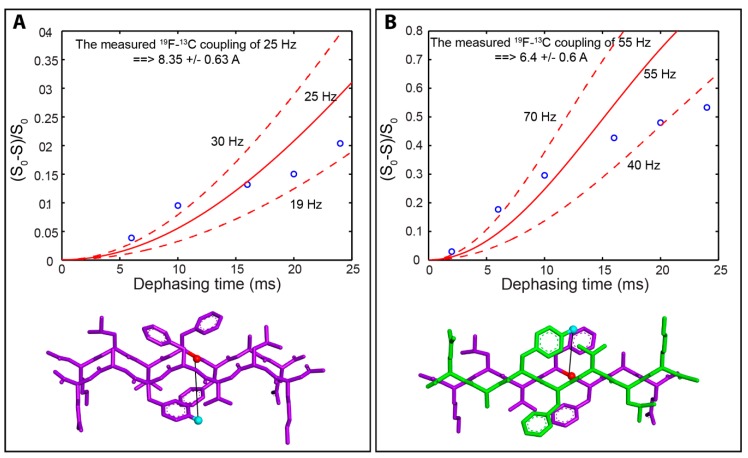
Dephasing curves for measurement of ^19^F-^13^C distance correlations: ((**A**). Dephasing curve depicted as ∆S/S_0_ vs. dephasing time (ms) used to determine the 1-^13^C to ^19^F distance in pleated β-sheet cofibrils of l-Ac-KLV**F**FAE-NH_2_ with l-Ac-KLVF(4-F-Phe)AE-NH_2_. At the bottom of the panel is a predictive model for the cross-strand β-sheet orientation of these peptides with the 1-^13^C label shown in red and the ^19^F label shown in cyan. The predicted ^19^F-^13^C distance is 8.63 Å and the measured ^19^F-^13^C distance is 8.4 Å. (**B**) Dephasing curve depicted as ∆S/S_0_ vs. dephasing time (ms) used to determine the 1-^13^C to ^19^F distance in rippled β-sheet cofibrils of l-Ac-KLV**F**FAE-NH_2_ with d-Ac-klvf(4-F-phe)ae-NH_2_. At the bottom of the panel is a predictive model for the cross-strand β-sheet orientation of these peptides in rippled β-sheets with the 1-^13^C label shown in red and the ^19^F label shown in cyan. The predicted ^19^F-^13^C distance is 6.48 Å and the measured ^19^F-^13^C distance is 6.4 Å.

Finally, we used solid-state nuclear magnetic resonance (ssNMR) spectroscopy to confirm formation of rippled β-sheets and to validate our models for the β-sheet packing structure for both pleated and rippled β-sheet assemblies. For these studies, we prepared Aβ(16–22) that contained either ^13^C or ^19^F labels to enable measurement of ^13^C–^19^F distances by ssNMR dipolar recoupling experiments. We synthesized three peptides for these experiments. The first was an l-Aβ(16–22) derivative with a 1-^13^C label at Phe 19: l-Ac-KLV**F**FAE-NH_2_, with the 1-^13^C-labeled Phe underlined and in bold. Next, we synthesized l- and d-Aβ(16–22) enantiomers with 4-F-phenylalanine in the Phe 20 position (l-Ac-KLVF(4-F-Phe)AE-NH_2_ and d-Ac-klvf(4-F-phe)ae-NH_2_). For these peptides, a ^19^F label was chosen for correlative cross-coupling experiments due to the lack of commercial sources for d-amino acids with ^13^C or ^15^N labels. The inclusion of a single F substituent on the Phe side chain is a conservative modification that does not significantly alter the assembly properties of the resulting Aβ(16–22) variants (see Figure S4). These peptides were assembled in two combinations: l-Ac-KLV**F**FAE-NH_2_ with l-Ac-KLVF(4-F-Phe)AE-NH_2_ and l-Ac-KLV**F**FAE-NH_2_ with d-Ac-klvf(4-F-phe)ae-NH_2_. The first combination, l-Ac-KLV**F**FAE-NH_2_ with l-Ac-KLVF(4-F-Phe)AE-NH_2_ was expected to coassemble into pleated β-sheet fibrils in which the peptides are statistically mixed, creating three possible pairs: ^13^C adjacent to ^13^C, ^13^C next to ^19^F, and ^19^F adjacent to ^19^F. When these peptides are aligned with a ^13^C strand next to a ^19^F strand within the fibrils, the ^13^C and ^19^F labels would be 8.63 Å apart according to our putative model for these assemblies (Figure 5A, bottom of panel). The second combination, l-Ac-KLV**F**FAE-NH_2_ with d-Ac-klvf(4-F-phe)ae-NH_2_, was predicted to coassemble into rippled β-sheets with the l- and d- peptides precisely aligned in an alternating pattern so that the ^13^C and ^19^F labels would be 6.48 Å apart based on our predictive model (Figure 5B bottom of panel).

The mixtures described above were coassembled to form cofibrils containing both ^13^C and ^19^F in cross-strand positions within the pleated or rippled β-sheet assemblies. The L-Ac-KLV**F**FAE-NH_2_ with L-Ac-KLVF(4-F-Phe)AE-NH_2_ mixture assembled into pleated β-sheet fibers that were indistinguishable from those shown in Figure 3A,B as shown in Figure S4. Likewise, the L-Ac-KLV**F**FAE-NH_2_ with d-Ac-klvf(4-F-phe)ae-NH_2_ mixture coassembled to form more rigid rippled β-sheet assemblies that were identical to those depicted in Figure 3C,D (Figure S4).

These pleated and rippled β-sheet fibrils were then subjected to ssNMR analysis to measure cross-strand ^13^C–^19^F distances in order to determine the accuracy of our models for the strand packing in pleated and rippled β-sheet assemblies. The labeled ^13^C and ^19^F peaks are readily observed by ssNMR in both the pleated and rippled β-sheet assemblies, facilitating the use of cross-polarization magic angle spinning (CP/MAS) techniques [33,34,35,36,37,38,39] to enhance the labeled ^13^C signal from protons and then to monitor the decay in the ^13^C signal intensity due to ^19^F dephasing (Figure 5A,B) through rotational echo double resonance (REDOR) spectroscopy [40]. Detailed protocols are provided in the Materials and Methods section. Since we observed the ^13^C signals (Figure S5, Supplemental Materials), two possible pairs (^13^C next to ^13^C and ^13^C next to ^19^F) contribute to the observed C13 signals. For the pleated β-sheet fibrils of l-Ac-KLVFFAE-NH_2_ with l-Ac-KLVF(4-F-Phe)AE-NH_2_, the dephasing data is shown in Figure 5A where the dashed lines represent the strongest and weakest possible ^19^F-^13^C dipolar couplings and the solid line was calculated using the ^19^F-^13^C dipolar coupling in between. The obtained distances and the error bars were obtained based on the strongest and weakest ^19^F-^13^C dipolar couplings. The data in Figure 5A indicates a dipolar coupling of 25 Hz, which corresponds to a ^13^C–^19^F distance of 8.4 ± 0.6 Å, consistent with the predicted distance of 8.63 Å. Note, the statistical assembly was taken into account in this experiment. As seen in Figure S5, there are two ^13^C peaks that reflect the two possible environments that this carbon can exist in based on the uncontrolled statistical assembly of the labeled peptides. Each REDOR point was obtained in two consecutive experiments, one without (S_0_) and one with (S) irradiation on the ^19^F channel. For the ^13^C-^13^C pair, there is no difference between the signal with and without the ^19^F irradiation. Thus, the ^13^C-^13^C signal is considered to be a background signal that does not contribute to the dipolar dephasing. Additionally, the ^19^F-^19^F pair does not affect the REDOR curves because we observed the ^13^C signals.

For the rippled β-sheet assemblies of l-Ac-KLVFFAE-NH_2_ with d-Ac-klvf(4-F-phe)ae-NH_2_, the dephasing data (Figure 5B) gave a dipolar coupling of 55 Hz, consistent with a ^13^C–^19^F distance of 6.4 ± 0.6 Å, very close to the predicted distance of 6.48 Å. Additionally, unlike the data obtained from the l/l- fibrils, the raw ssNMR data obtained from the l/d- sample was very sharp with no shoulders as shown in Figure S5, indicating a single packing pattern of ^13^C next to ^19^F, and confirming the formation of rippled β-sheets with strictly alternating l/d packing structure in enantiomeric mixtures of l- and d-Aβ(16–22) as opposed to statistical mixtures as in the l-Ac-KLVFFAE-NH_2_ with l-Ac-KLVF(4-F-Phe)AE-NH_2_. These data are also consistent with the predictive models for pleated and rippled β-sheets that indicate an “eclipsed”-like arrangement of side chain cross-strand packing in pleated β-sheets and a “staggered”-like arrangement of side chains in rippled β-sheets. These differences in packing structure most likely account for the relative energetic stability of rippled β-sheets.

## 3. Conclusions

In conclusion, previous reports have shown that enantiomeric amphipathic peptides with alternating hydrophilic and hydrophobic side chains readily coassemble into rippled β-sheets, as predicted by Pauling and Corey [13,17,18,19,20]. Here, we have demonstrated that this phenomenon is not restricted to β-strand peptides with this particular sequence pattern but can be extended to the peptide Aβ(16–22), which has a nonpolar core and polar termini. The results reported herein also indicate that rippled β-sheet formation via coassembly of enantiomeric peptides is kinetically and thermodynamically favorable relative to the self-assembly of the corresponding individual enantiomers. The basis for these preferences is not yet completely understood, but is likely due to the altered cross-strand packing structure in rippled β-sheets compared to pleated β-sheets, which has been recently shown to adopt a “staggered” side chain conformations (rippled sheets), as opposed to “eclipsed” side chain conformations (pleated sheets) [19]. The extension of rippled β-sheet formation suggested by the study reported herein is also consistent with recent work that showed that neurotoxic species were avoided when full-length l-Aβ_42_ and D-Aβ_42_ were mixed, resulting in unique aggregates suggestive of rippled β-sheets [29]. Also, cross-seeding of poly-glutamine amyloid by seeds of the opposite enantiomer suggest that rippled β-sheets are also accessible by this amyloid species [41]. However, there are some examples of enantiomeric mixtures of self-assembling peptides that do not successfully coassemble, and instead self-sort. Recent work has shown that disulfide bound dimers of peptides derived from β2-microglobulin self-sort and that enantiomeric mixtures of full length β2-microglobulin mostly self-sort, with perhaps a small amount of coassembly occurring [42]. From this study, it is apparent that the ability of enantiomeric β-sheet peptides to undergo coassembly is not straightforward, and thus invites further study. More detailed high-resolution structural analyses of rippled β-sheet materials, including the Aβ(16–22) materials described herein, will provide additional insight into the preference for enantiomeric rippled β-sheet assembly and will facilitate the design of novel rippled β-sheet materials and strategies whereby these mechanisms might be exploited to divert amyloid assembly in vivo into non-destructive pathways.

## 4. Materials and Methods

### 4.1. Peptide Synthesis

All peptides were synthesized using standard solid-phase techniques with Fmoc protection and HBTU/HOBt or DIC/HOBt activation on Rink amide resin (Advanced ChemTech, 100–200 mesh, 0.27 mmol/g, Louisville, KY, USA) as *N*-acetyl, *C*-terminal amide sequences. After Fmoc deprotection of the final amino acid, the *N-*termini were acetylated followed by sidechain deprotection and cleavage from the resin using a 95:2.5:2.5 *v/v* trifluoroacetic acid:triisopropylsilane:water solution. The resulting solution was concentrated and cold diethyl ether was added to initiate precipitation of the crude peptide product. The precipitate was pelleted out via centrifugation and dissolved in DMSO prior to purification by preparatory HPLC.

### 4.2. Peptide Purification and Characterization

Peptide purification was performed using a reverse phase Phenomenex Gemini (5 μm, NX-C18, 110 Å, 250 × 21.2 mm, Phenomenex, Torrance, CA, USA) column on a Shimadzu LC 6-AD HPLC (Shimadzu Corporation, Nakagyo-ku, Kyoto, Japan) with a binary gradient of water and acetonitrile with 0.1% TFA at 10 mL min^−1^. UV absorbance at 215 and 254 nm of the eluent was monitored for fraction collection. Peptide purity was confirmed using MALDI-TOF mass spectroscopy and analytical HPLC using a reverse phase Phenomenex Gemini (5 μm, C18, 110 Å, 250 × 4.6 mm, Phenomenex, Torrance, CA, USA) column on a Shimadzu LC-2010A (Shimadzu Corporation, Nakagyo-ku, Kyoto, Japan). Following purification, purified peptide fractions were lyophilized prior to disaggregation and assembly.

### 4.3. Peptide Disaggregation Protocol

Peptide disaggregation was performed using a modified Wetzel protocol [28]. Lyophilized, purified peptide samples were dissolved in 2 mL TFA and sonicated for 10 min. After sonication, TFA was evaporated under a gentle stream of nitrogen and the resulting peptide film reconstituted in 2 mL HFIP and incubated at 37 °C for 90 min. Following incubation, the HFIP was evaporated under a gentle stream of nitrogen and the resulting peptide film again taken up in 2 mL HFIP. Peptide concentration was then checked by diluting 10 μL peptide solution in DMSO and analyzing via analytical HPLC for comparison to a concentration curve calibrated by amino acid analysis. Peptide solution was then aliquoted appropriately into lo-bind Eppendorf tubes and dried in vacuo for 16 h.

### 4.4. Sedimentation Assays

Sedimentation assays were performed using a modified Wetzel protocol [28]. Dried, disaggregated peptide aliquots were dissolved in DMSO and diluted into the desired amount of phosphate buffered saline (137 mM NaCl, 2.7 mM KCl, 10 mM Na_2_HPO_4_, 1.8 mM KH_2_PO_4_, pH 7.4, 0.1% NaN_3_ *w*/*v*; DMSO was 5% by volume of the final solution) in glass vials. For coassembly samples, l- and d- peptides were kept separate until dilution into PBS. Samples were incubated at 37 °C and 250 μL aliquots were removed at predetermined times. Aliquots were then centrifuged at 436,000 × *g* for 1 h at 4 °C to remove oligomeric and fibrillar aggregates. After centrifugation, the supernatant was collected and diluted with DMSO to halt further assembly. These solutions were then analyzed by analytical HPLC for correlation to a concentration curve to determine remaining monomer concentration.

### 4.5. Solid-State Fourier Transform Infrared Spectroscopy (FTIR)

Dried, disaggregated peptide aliquots were prepared for assembly as in sedimentation assays, but in larger volumes (greater than or equal to 5 mL) and were allowed to assemble for 21 days so as to allow for complete assembly of all samples. Following assembly, samples were centrifuged at 4300 × *g* for 90 min at 17 °C to collect aggregates. The supernatant was then carefully removed and deionized water added, mixed, and centrifugation repeated to wash. After first wash, supernatant was again removed and deionized water added and mixed, at which point samples were spotted for TEM or SEM if desired (below). Centrifugation was repeated once more and supernatant removed, and then samples were frozen and lyophilized. Lyophilized pellets were then placed on the solid-state stage of a Shimadzu FTIR-8400S (Shimadzu Corporation, Nakagyo-ku, Kyoto, Japan) spectrometer and analyzed at a resolution of 4 cm^−1^.

### 4.6. Transmission Electron Microscopy (TEM)

Fibrils for the TEM images reported were obtained from the same samples that were used for FTIR, above, at the same timepoint. Fibrils were washed as described above and 5 μL aliquots of the resulting fibril solutions in water were applied to TEM grids (carbon film coated copper, 200 mesh; Electron Microscopy Sciences, Hatfield, PA, USA) and allowed to adsorb for 30 s. The excess fluid was removed by capillary action and then 5 μL 5% uranyl acetate was allowed to adsorb on the grid for 2 min to stain the sample. Excess staining solution was removed by capillary action and grids were allowed to dry in air for 10 min. TEM images were recorded on a Hitachi 7650 transmission electron microscope in high contrast mode with an accelerating voltage of 80 kV. Fibril widths were measured in ImageJ and are reported as averages from 100–150 measurements.

### 4.7. Scanning Electron Microscopy (SEM)

Fibrils were washed as described above and 5 μL aliquots of the resulting fibril solutions in water were applied to round 12 mm coverglasses and allowed to dry. Coverglasses were then mounted onto aluminum stubs, sputter coated with gold, and imaged using a Zeiss Auriga Field Emission Scanning Electron Microscope (Zeiss International, Oberkochen, Germany).

### 4.8. Solid State Nuclear Magnetic Resonance (ssNMR) Spectroscopy

Dried, disaggregated peptide aliquots were prepared for assembly as in sedimentation assays, but in large volumes (90 mL) at high concentrations of total peptide (0.78 mM) and were allowed to assemble for 8 days prior to collection and washing as described in FTIR sample prep. Lyophilized materials were analyzed at the National High Magnetic Field Lab (Tallahassee, Florida, USA).

Solid state NMR experiments were performed on a Bruker Avance 600 MHz NMR spectrometer (Bruker Corporation, Billerica, MA, USA) with a Bruker HFX triple-resonance MAS NMR probe (Bruker Corporation, Billerica, MA, USA) and equipped with a Bruker pneumatic MAS unit (Bruker Corporation, Billerica, MA, USA). Samples were spun at 13 kHz ± 3 Hz. The Larmor frequencies of ^1^H, ^19^F, and ^13^C are 600.13, 564.64, and 150.91 MHz, respectively. ^13^C magnetization was enhanced from ^1^H through cross polarization using a contact time of 1.5 ms, during which a ^1^H spin-lock field of 50 kHz was used and the ^13^C B_1_ field was ramped from 45 to 65 kHz. The rotor synchronized spin-echo pulse sequence was used to record ^13^C signals. A ^13^C 180° pulse was applied on the chemical shift position of ~175 ppm in the middle of the spin-echo time. Two ^19^F 180° pulses per rotor period were applied during the spin-echo time (or dephasing time) in order to dephase the ^13^C magnetization due to the recovery of the ^13^C-^19^F dipolar coupling. The ^19^F 180° pulse length was 6.0 µs. The XY-8 pulse sequence [43] was applied to the ^19^F 180° pulse trains in order to compensate for the flip angle error and the off-resonance effects. A SPINAL64 decoupling sequence [44] with a ^1^H B_1_ field of 62.5 kHz was used during the spin-echo time and acquisition. A MATLAB program was used to calculate the dephasing curves at various distances. The ^13^C chemical shift was referenced to the carbonyl carbon resonance of glycine at 178.4 ppm relative to TMS.

## Supplementary Materials

The following are available online. Table S1: HPLC gradient conditions, Figure S1: analytical HPLC traces, Table S2: Calculated and observed *m*/*z* values (MALDI-TOF-MS), Figure S2: MALDI-TOF-MS spectra, Figure S3: Peptide concentration curves, Figure S4: Additional TEM images, Figure S5: ssNMR one-dimensional ^13^C spectra.
