# Supplementary material for: Rippled β-Sheet Formation by an Amyloid-β Fragment Indicates Expanded Scope of Sequence Space for Enantiomeric β-Sheet Peptide Coassembly
Source: Molecules. 2019 May 23;24(10):1983. doi: 10.3390/molecules24101983 (PMC6571685; doi:10.3390/molecules24101983)
Supplement: Supplementary file 1 [file molecules-24-01983-s001.pdf]

Rippled β-sheet formation by an amyloid-β fragment suggests the generality of enantiomeric β-sheet peptide coassembly

*Jennifer M. Urban,[1] Janson Ho,[1] Gavin Piester,[1] Riqiang Fu,[2] and Bradley L. Nilsson[1]**

[1]Department of Chemistry, University of Rochester, Rochester, NY 14627-0216

[2]The National High Magnetic Field Laboratory, Florida State University, 1800 East Paul Dirac Drive, Tallahassee, FL 32310

email: bradley.nilsson@ rochester.edu; Fax: (585) 276-0205; Tel: (585) 276-3053

SUPPLEMENTARY MATERIAL

Contents:

Table S1. Analytical HPLC gradient conditions …………………………….……….... page S2
Figure S1. Analytical HPLC traces ………………….….……………………........... page S2-4
Table S2. Calculated and observed *m/z* values (MALDI-TOF-MS)…………….…page S4
Figure S2. MALDI-TOF-MS spectra……………….……………………….….…page S5-7
Figure S3. Peptide concentration curves..…………………………………………page S8
Figure S4. TEM images of peptide fibrils used for solid state NMR..…..………...page S9-S10
Figure S5. Raw ssNMR data for the L/L- and L/D- assemblies……………………page S11

Table S1. Analytical HPLC gradient conditions

Peptide	Sequence	Retention Time (min)	Gradient (soln A: water/0.5% TFA; soln B: acetonitrile/0.5% TFA)
L-Aβ(16-22)	Ac-KLVFFAE-NH_2	12.55	Isocratic 5% **B** 5 min, 5-95% **B** over 10 min, 95% **B** 5 min
D-Aβ(16-22)	Ac-klvffae-NH_2	12.55	Isocratic 5% **B** 5 min, 5-95% **B** over 10 min, 95% **B** 5 min
L17/F20 ^{13}C L-Aβ(16-22)	Ac-K^{13}LVF^{13}FAE-NH_2	12.55	Isocratic 5% **B** 5 min, 5-95% **B** over 10 min, 95% **B** 5 min
F19 ^{13}C L-Aβ(16-22)	Ac-KLV^{13}FFAE-NH_2	12.35	Isocratic 5% **B** 5 min, 5-95% **B** over 10 min, 95% **B** 5 min
F20 4F-Phe L-Aβ(16-22)	Ac-KLVF(4F-Phe)AE-NH_2	12.43	Isocratic 5% **B** 5 min, 5-95% **B** over 10 min, 95% **B** 5 min
F20 D-4F-phe D-Aβ(16-22)	Ac-klvf(4F-phe)AE-NH_2	12.43	Isocratic 5% **B** 5 min, 5-95% **B** over 10 min, 95% **B** 5 min

Figure S1. Analytical HPLC traces of synthetic peptides at 215 nm.

A. L-Aβ(16-22), Ac-KLVFFAE-NH_2

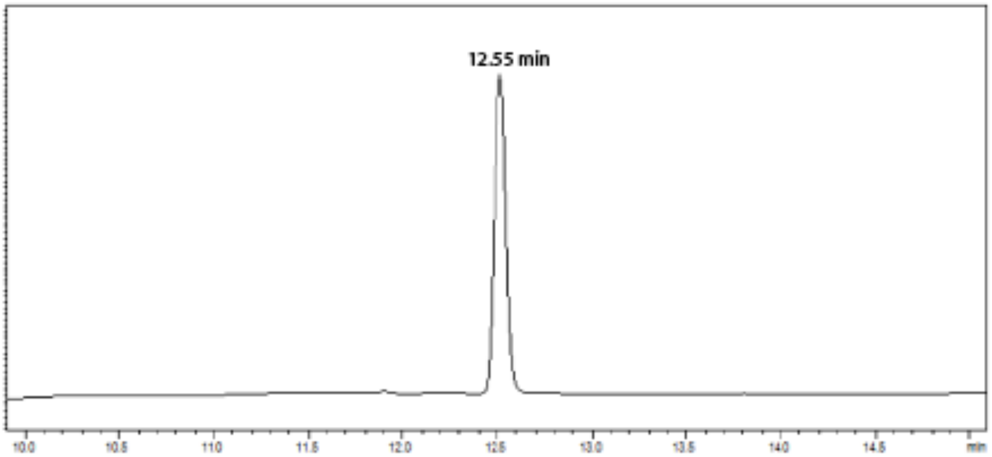

B. D-Aβ(16-22), Ac-klvffae-NH$_2$

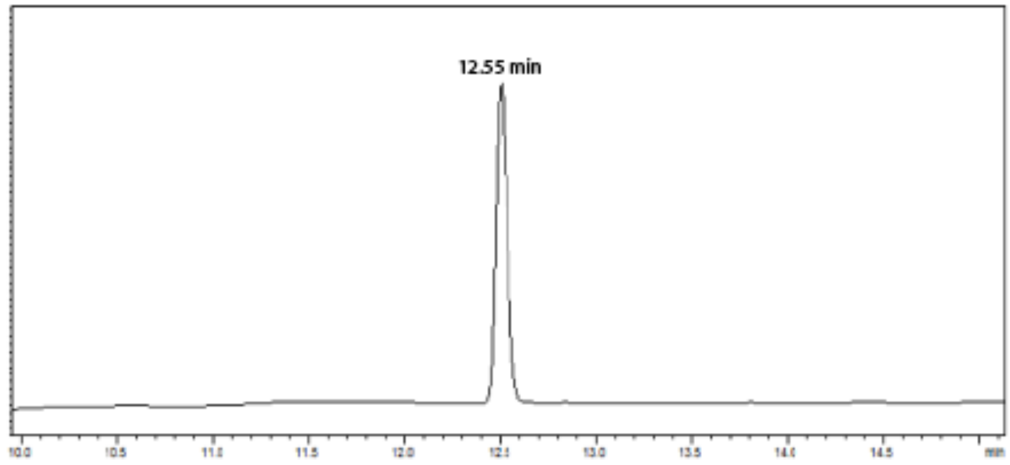

C. L17/F20 ^{13}C L-Aβ(16-22), Ac-K^{13}LVF^{13}FAE-NH$_2$

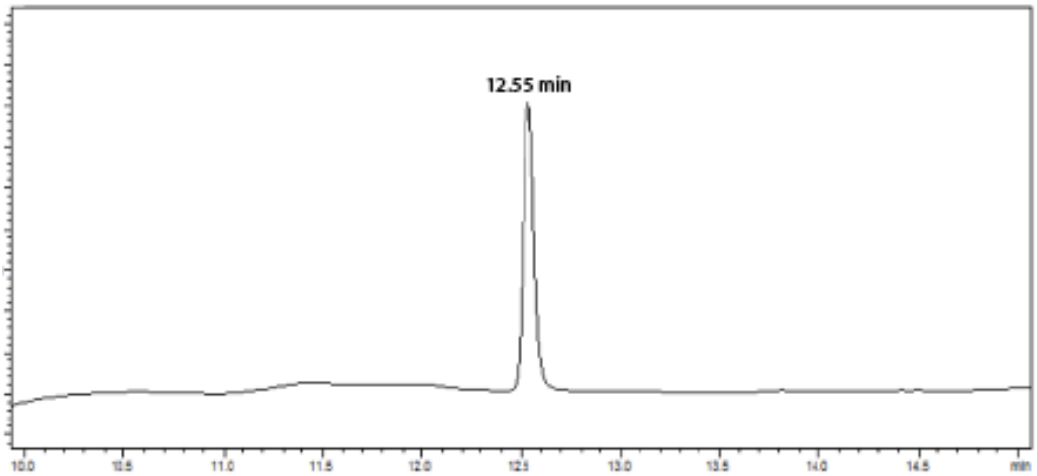

D. F19 ^{13}C L-Aβ(16-22), Ac-KLV^{13}FFAE-NH$_2$

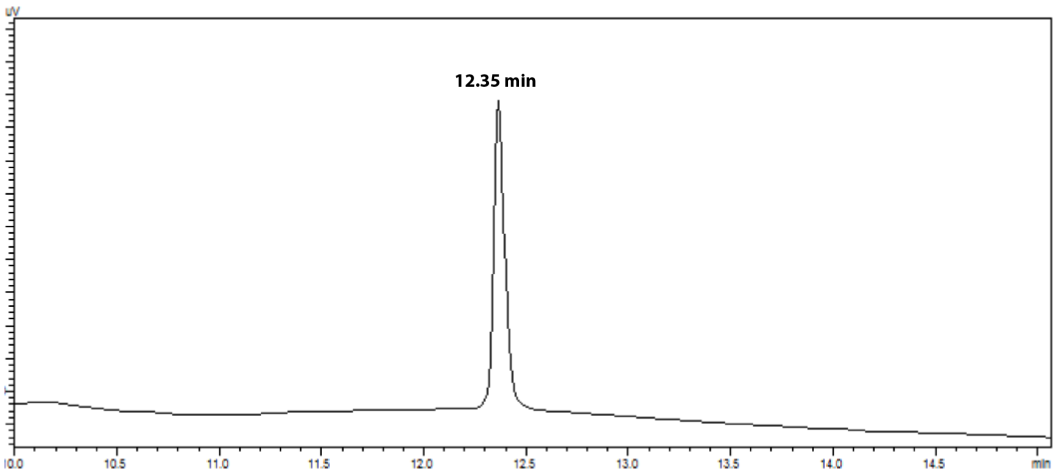

E. F20 4F-Phe L-Aβ(16-22), Ac-KLVF(4F-Phe)AE-NH$_2$

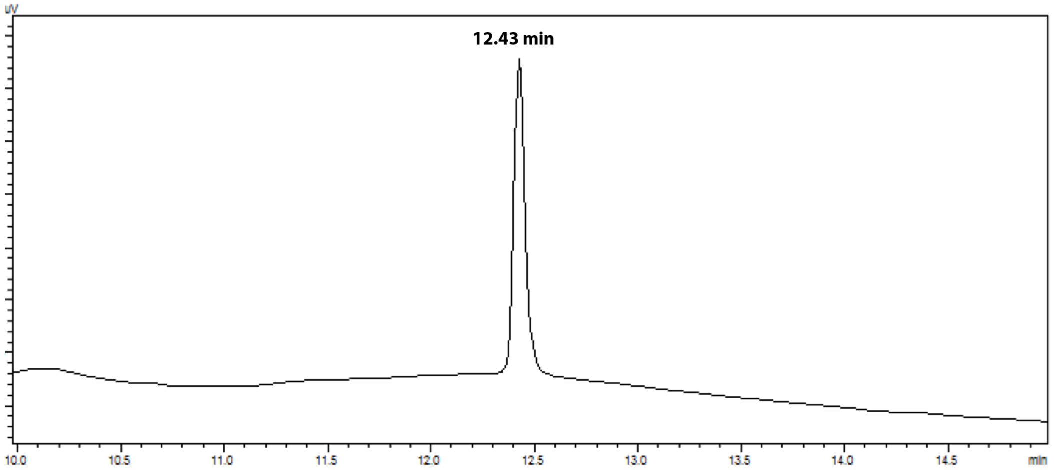

F. F20 D-4F-phe D-Aβ(16-22), Ac-klvf(4F-phe)AE-NH$_2$

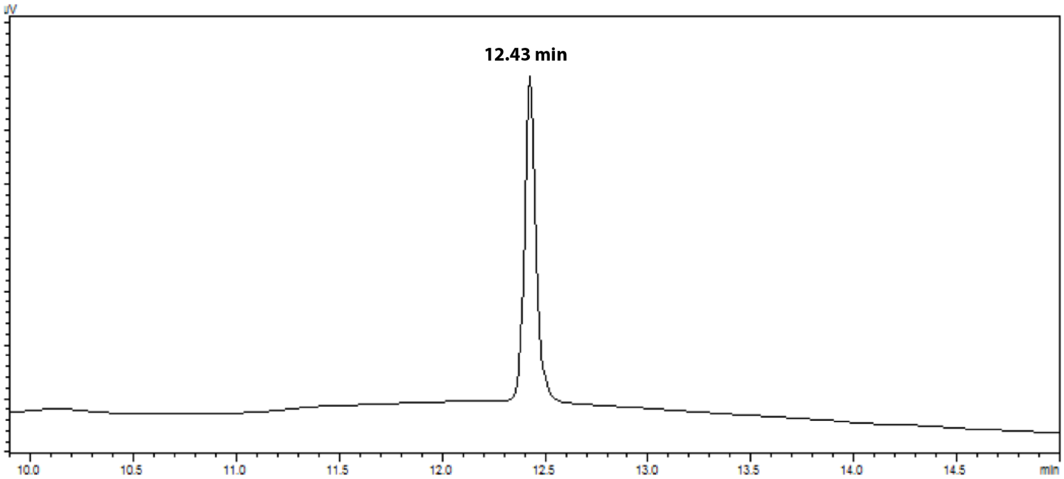

Table S2. Calculated and observed *m*/*z* for all peptides by MALDI-TOF-MS.

Peptide	calc [MH$^+$]	obs [MH$^+$]	calc [MNa$^+$]	obs [MNa$^+$]	calc [MK$^+$]	obs [MK$^+$]
L-Aβ(16-22)	895.07	895.175	917.07	917.191	933.17	933.180
D-Aβ(16-22)	895.07	894.693	917.07	916.685	933.17	932.671
L17/F20 ^{13}C L-Aβ(16-22)	897.05	896.834	919.05	918.835	935.15	934.821
F19 ^{13}C L-Aβ(16-22)	896.06	896.311	918.06	918.340	934.16	934.335
F20 4F-Phe L-Aβ(16-22)	913.06	913.494	935.06	935.522	951.16	951.523

F20 D-4F-phe D-Aβ(16-22)	913.06	913.497	935.06	935.532	951.16	951.530

Figure S2. MALDI-TOF spectra for all peptides.

A. L-Aβ(16-22), Ac-FKLVFFAE-NH$_2$

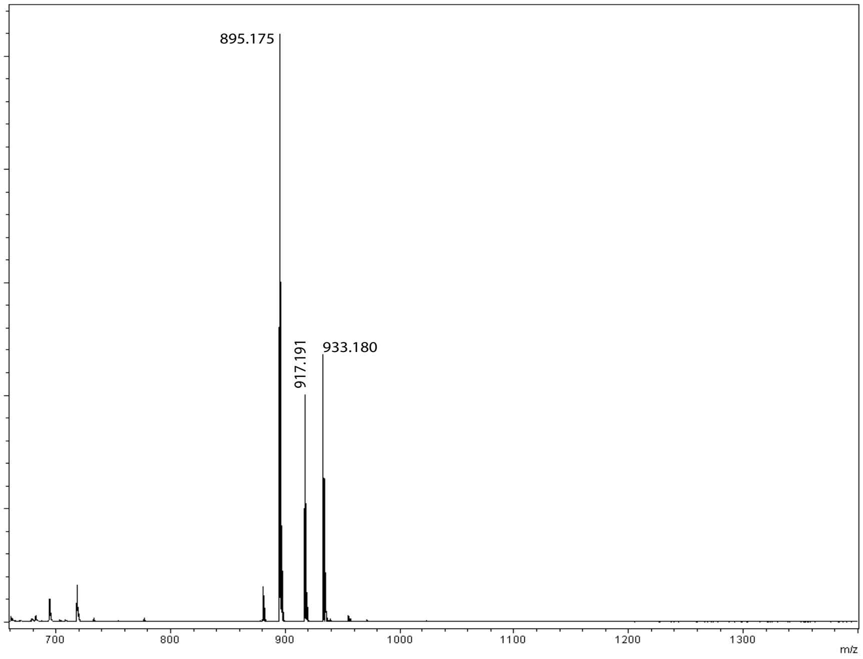

B. D-Aβ(16-22), Ac-klvffae-NH$_2$

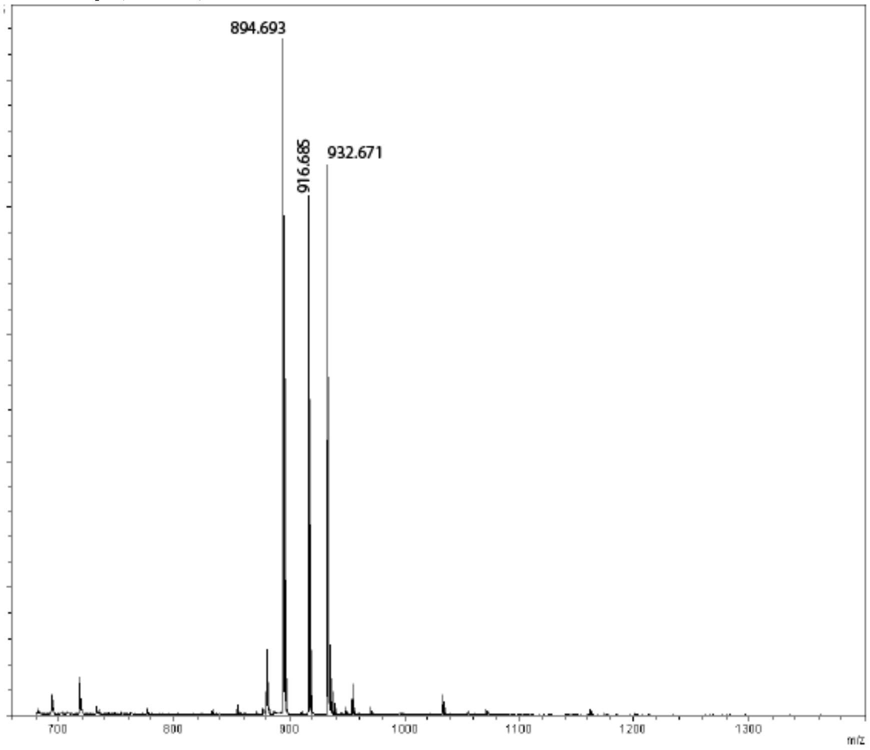

C. L17/F20 ^{13}C L-Aβ(16-22), Ac-K^{13}LVF^{13}FAE-NH$_2$

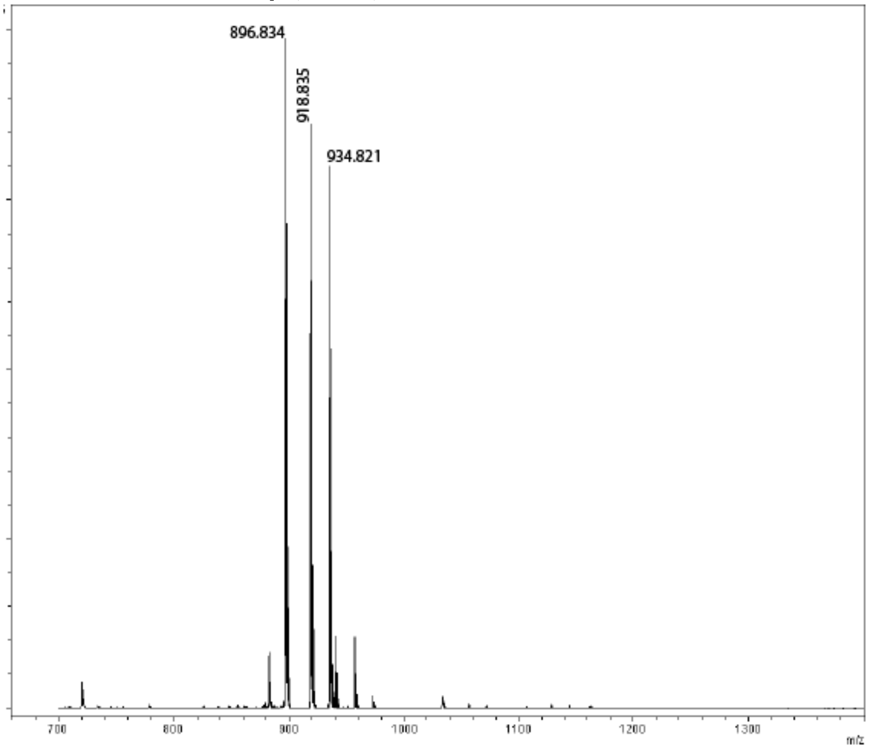

D. F19 ^{13}C L-Aβ(16-22), Ac-KLV^{13}FFAE-NH$_2$

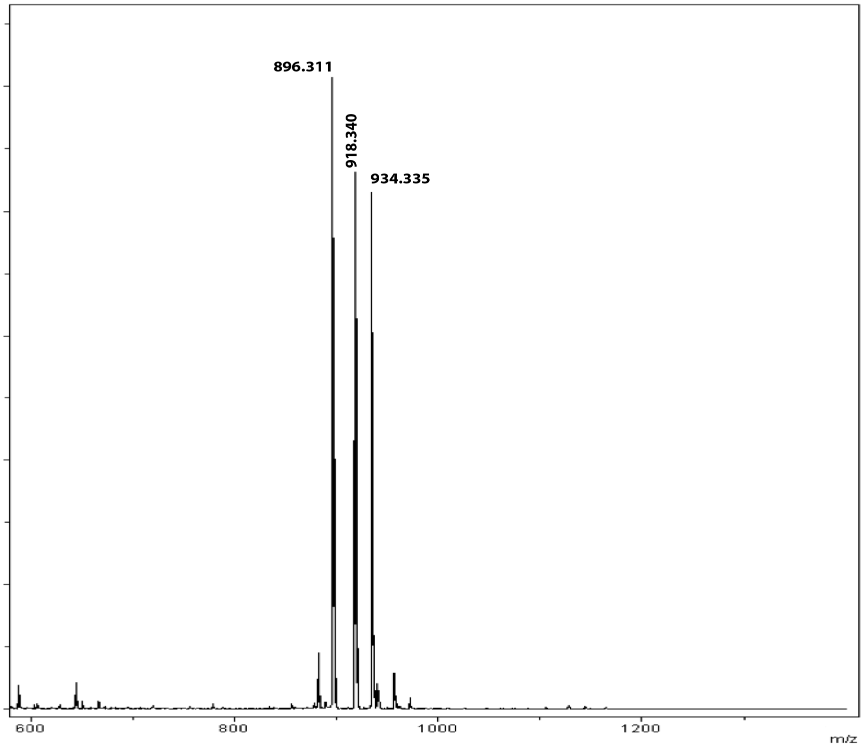

E. F20 4F-Phe L-Aβ(16-22), Ac-KLVF(4F-Phe)AE-NH$_2$

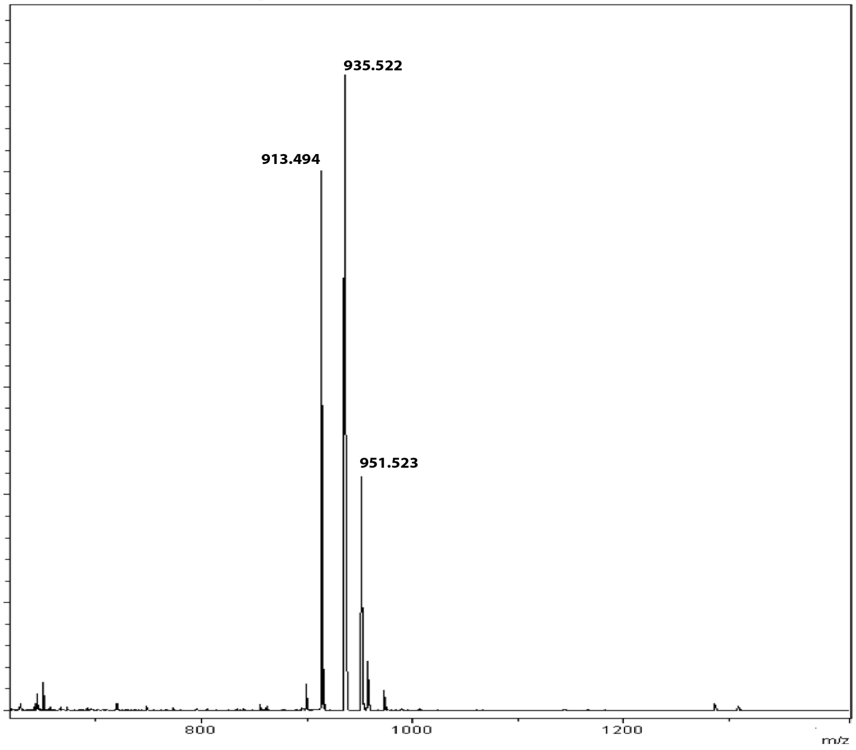

F. F20 D-4F-phe D-Aβ(16-22), Ac-klvf(4F-phe)AE-NH$_2$

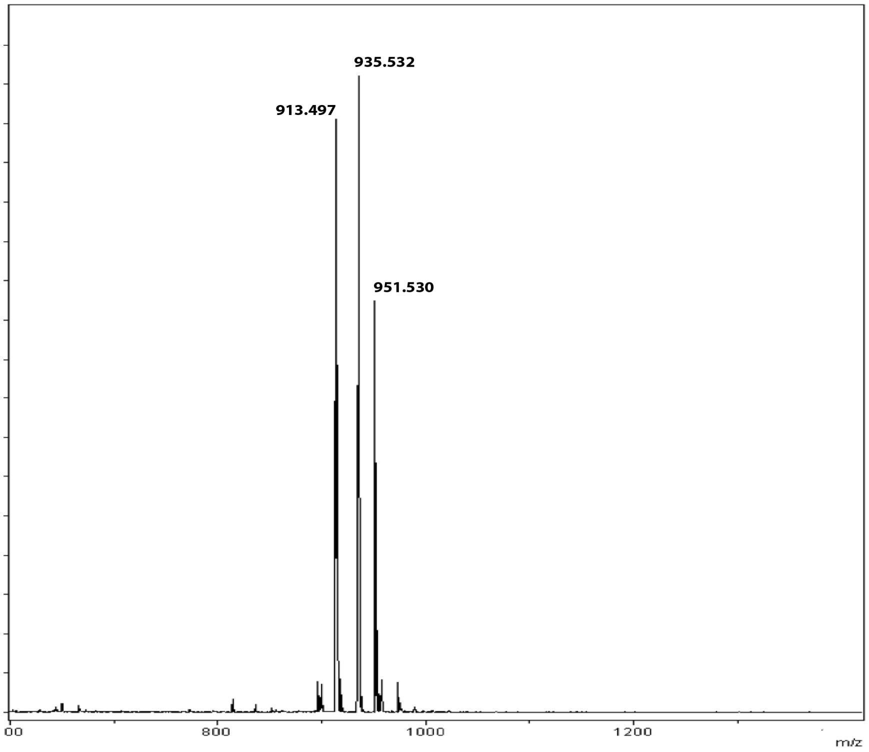

Figure S3. Peptide concentration curves used to determine concentrations of all peptides used in this study.

A. Concentration curve used for all peptides *except* those containing 4F-Phe

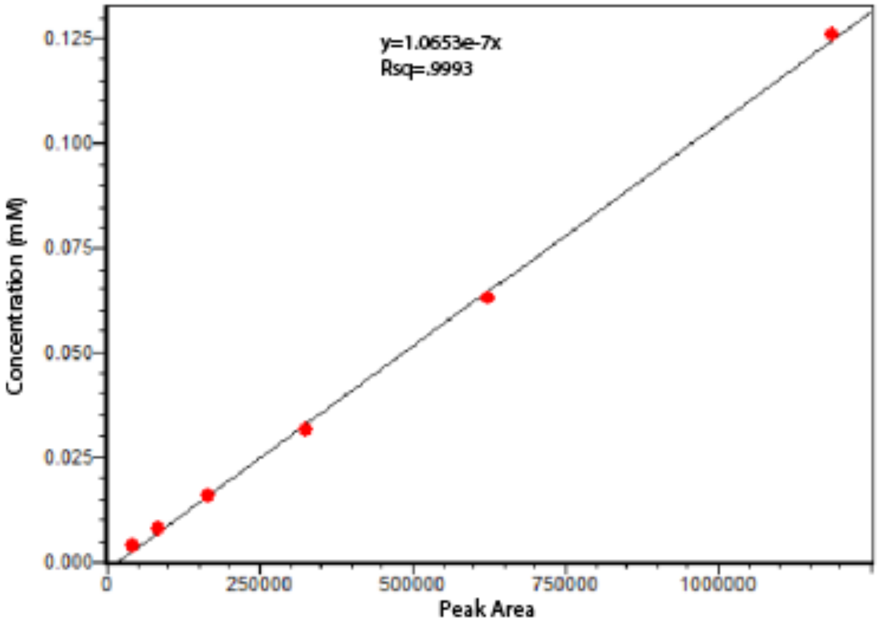

B. Concentration curve used for peptides containing 4F-Phe

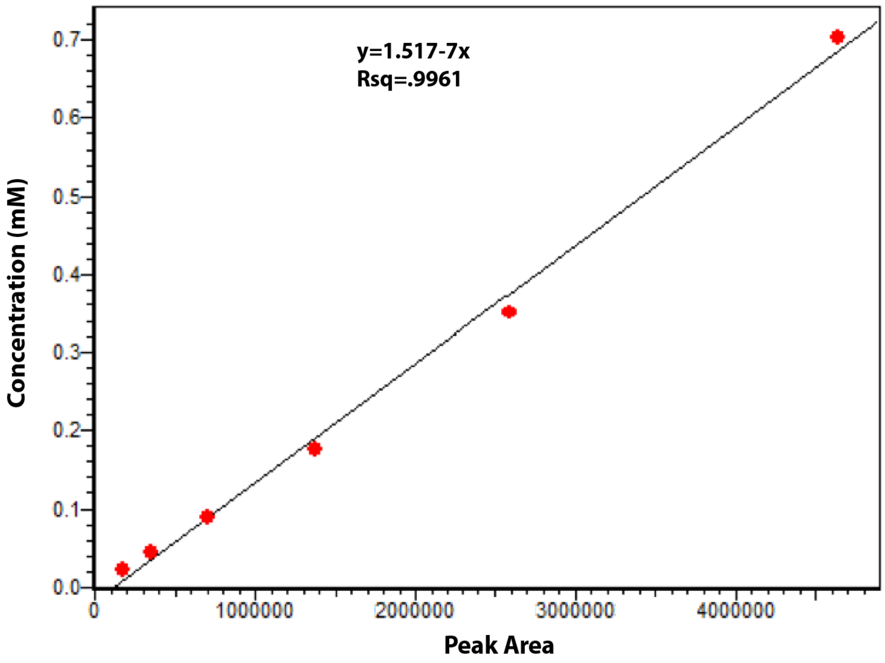

Figure S4. Transmission electron micrographs of peptide fibrils used for solid state NMR.

A. F20 4F-Phe L-Aβ(16-22), Ac-KLVF(4F-Phe)AE-NH$_2$

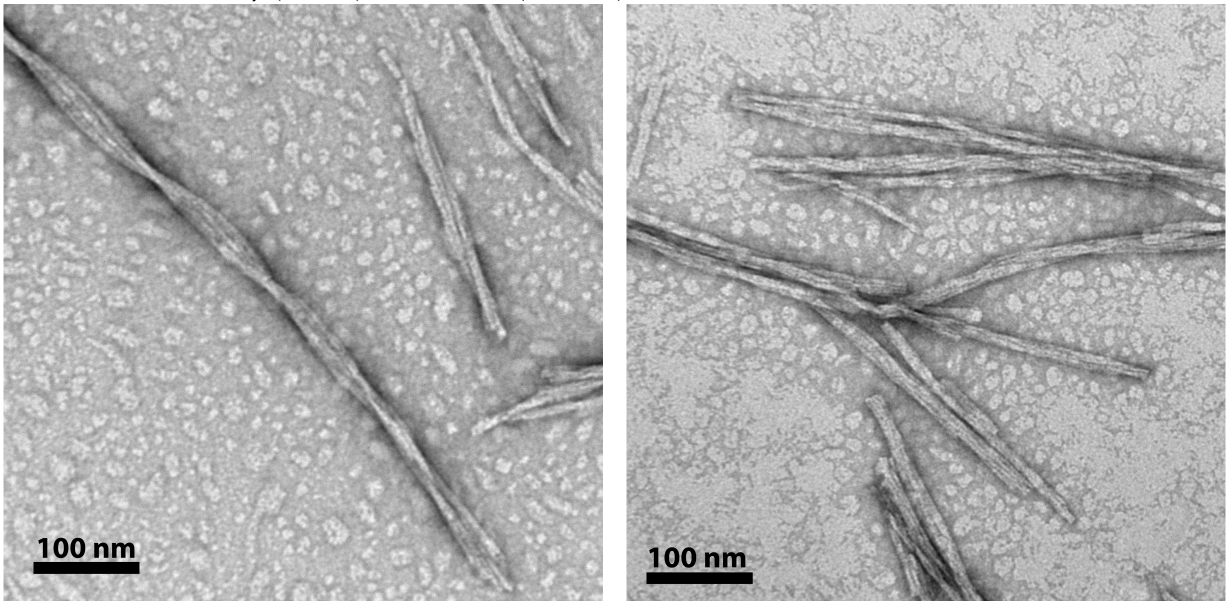

B. TEM images of the L/L-ssNMR sample: F19 ^{13}C L-Aβ(16-22) (Ac-KLV^{13}FFAE-NH$_2$) with F20 4F-Phe L-Aβ(16-22) (Ac-KLVF(4F-Phe)AE-NH$_2$).

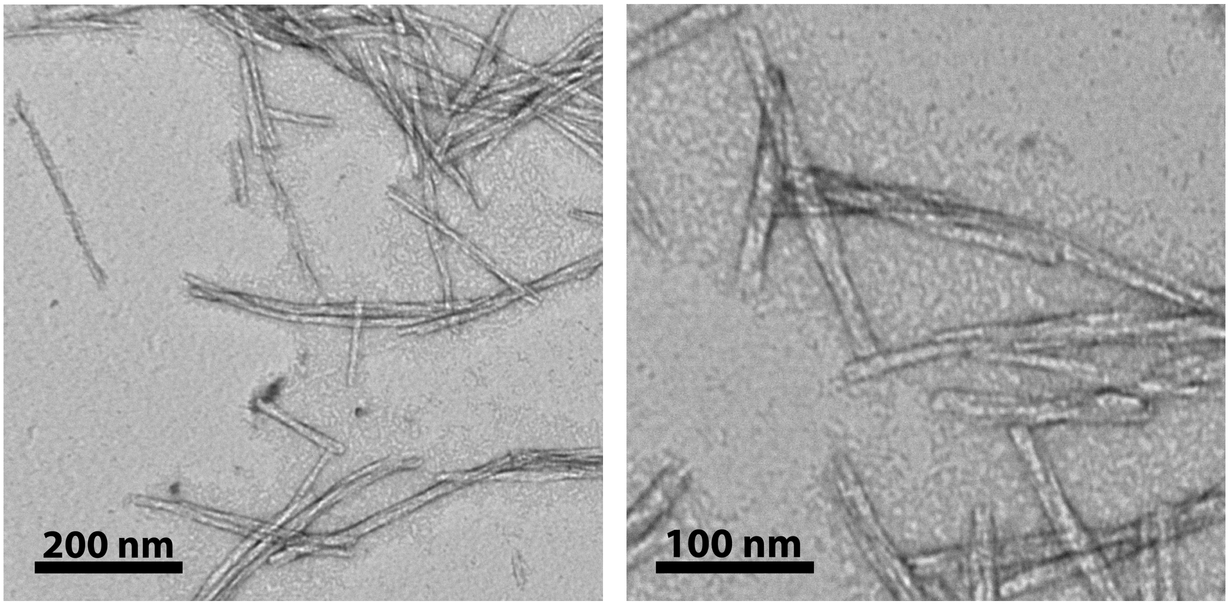

C. TEM images for the L/D-ssNMR sample: F19 ^{13}C L-Aβ(16-22) (Ac-KLV^{13}FFAE-NH$_2$) with F20 D-4F-phe D-Aβ(16-22) (Ac-klvf(4F-phe)AE-NH$_2$).

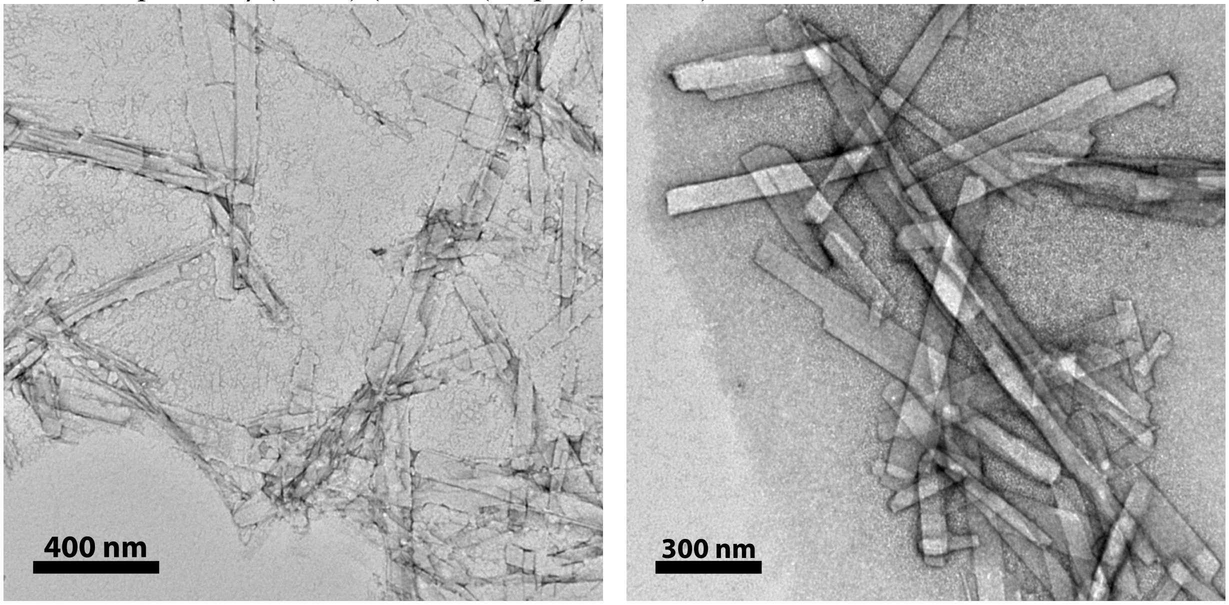

Figure S5. Carbon-13 ssNMR 1D spectra for the L/L- and L/D- Aβ(16–22) assemblies.

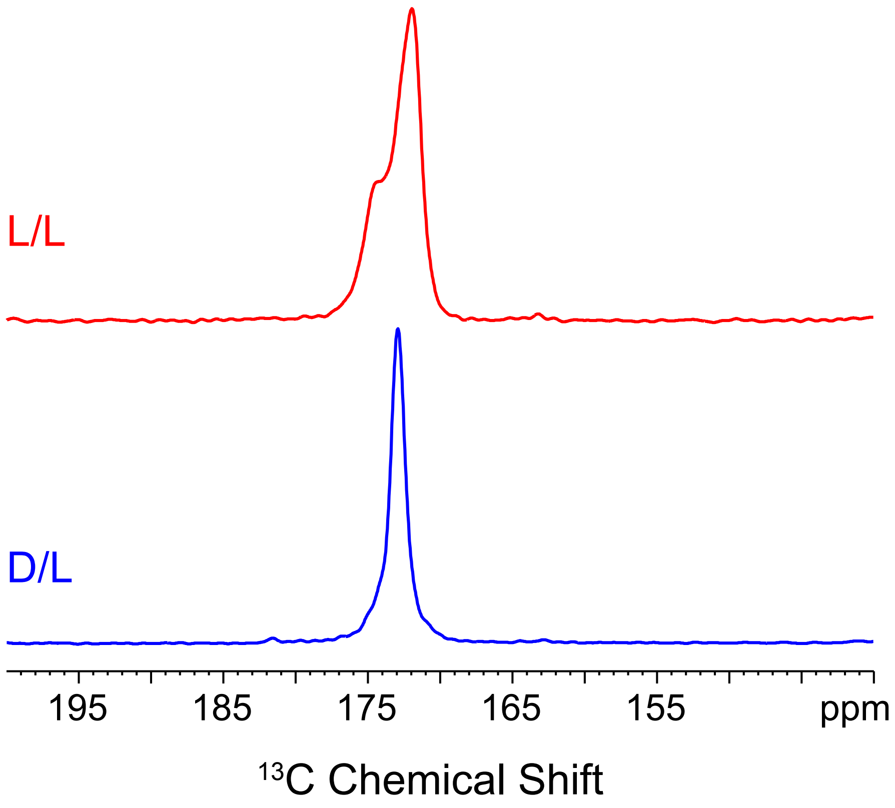
L/L
D/L
195
185
175
165
155
ppm
13C Chemical Shift
